# Canine and feline cardiopulmonary parasitic nematodes in Europe: emerging and underestimated

**DOI:** 10.1186/1756-3305-3-62

**Published:** 2010-07-23

**Authors:** Donato Traversa, Angela Di Cesare, Gary Conboy

**Affiliations:** 1 Department of Comparative Biomedical Sciences, University of Teramo, Teramo, Italy; 2 Department of Pathology and Microbiology, Atlantic Veterinary College, Charlottetown, PEI, Canada

## Abstract

Cardiopulmonary nematodes of dogs and cats cause parasitic diseases of central relevance in current veterinary practice. In the recent past the distribution of canine and feline heartworms and lungworms has increased in various geographical areas, including Europe. This is true especially for the metastrongyloids *Aelurostrongylus abstrusus*, *Angiostrongylus vasorum *and *Crenosoma vulpis*, the filarioid *Dirofilaria immitis *and the trichuroid *Eucoleus aerophilus *(syn. *Capillaria aerophila*). The reasons of this emergence are little known but many drivers such as global warming, changes in vector epidemiology and movements in animal populations, may be taken into account. The purpose of this article is to review the knowledge of the most important heartworm and lungworm infections of dogs and cats in Europe. In particular recent advances in epidemiology, clinical and control are described and discussed.

## Background

Nematodes affecting the cardiopulmonary system of dogs and cats have recently become the focus of increased attention from the scientific community due to their emergence in several European countries and the spread into previously non-endemic regions. This has been particularly the case for the metastrongyloids *Aelurostrongylus abstrusus*, *Angiostrongylus vasorum *and *Crenosoma vulpis*, the filarioid *Dirofilaria immitis *and the trichuroid *Eucoleus aerophilus *(syn. *Capillaria aerophila*). Indeed, the importance of infection with the heartworm (*A. vasorum *and *D. immitis*) and lungworm (*A. abstrusus*, *C. vulpis *and *E. aerophilus*) parasites in companion animals is heightened by the pathogenic potential of these nematodes, the challenges involved in diagnosis and (for some) their zoonotic potential.

The reasons for the apparent emergence of cardiopulmonary parasitoses in pets are unknown but several factors such as global warming, changes in vector seasonal population dynamics and movements in animal populations, may play a role in the recent rise in reports of infection in the various countries of Europe. Most of these parasites have an indirect life cycle, thus requiring an intermediate host (i.e. biological vector) for their development. This is true for the gastropod-borne *A. abstrusus*, *A. vasorum *and *C. vulpis *and for the mosquito-transmitted *D. immitis*. Conversely, *E. aerophilus *may develop either directly in the environment or in earthworms acting as facultative intermediate hosts. Consequently, the likelihood for a dog or a cat becoming infected by a cardiopulmonary nematode depends not only on the presence of the vector(s) but also by their abundance and the prevalence of the infection. The occurrence of canine and feline heartworms and lungworms in different geographical areas is mainly influenced by the presence of competent gastropod and culicid species, with the exception of *E. aerophilus*, whose presence is guaranteed by the ubiquity of the earthworms and by the direct life cycle. Therefore, the interaction between the parasites, their hosts and the environment plays a central role in the apparent increase in exposure risk of these infections and is the basis for a new recognition of the relative importance of these parasites as aetiological agents in cardiopulmonary disease in companion animals. As a consequence, in the past few years several publications mainly related to distribution, epidemiology and control have appeared, although several aspects on biology, pathology and diagnostics still need to be elucidated. Therefore the present paper aims to review the current knowledge on cardiopulmonary nematodes of dogs and cats with a focus on their epidemiological patterns in Europe, on the pathogenic impact they have and on the new avenues and perspectives for diagnosis and control.

## Distribution in Europe: changing patterns, certainties and dilemmas

The development and survival of the gastropod and insect vectors is mainly influenced by temperature, moisture and water availability, thus one of the most important factors currently favouring the dispersal and spread of vector-borne pathogens is global warming [1]. Specifically, given that rates of physiological processes in most invertebrates depend greatly on environmental temperature, the distribution and development rates of vector-borne diseases would be greatly influenced by a rise in temperature [2,3].

These environmental changes may play a causative role in the expansion of the geographic distribution of the heartworms, *D. immitis *("canine heartworm") and *A. vasorum *("French heartworm"). Population dynamics and activity of culicid and gastropod intermediate hosts are highly sensitive to temperature and moisture, and the development of nematode larvae in vectors is also known to be temperature-dependent [4]. Both *D. immitis *and *A. vasorum *have a huge range of competent vector species and their canine definitive hosts are distributed worldwide. As a likely consequence of these factors, the most recent papers report a trend in the geographic spread of both of the heartworms, *D. immitis *and *A. vasorum*, into previously free areas [5-9]. Transmission of *D. immitis *by mosquitoes is strictly dependant on a suitable climate allowing the larval development in the vectors, thus environmental temperatures are the main factors favoring the life cycle of this parasite [5,10,11]. Some climate-based models have been developed to investigate the possible changes that may occur in terms of distribution and seasonality for *D. immitis *in response to global warming [5,12,13]. These studies clearly demonstrated that *Dirofilaria *infections in Europe have seasonal transmission patterns with peak activities in the summer months and that warm climates facilitate larval development in the intermediate hosts. Therefore, changes in climate are likely to have a strong impact on parasite distribution, development and transmission patterns. The current trend of increasing temperatures will probably allow the spread of the nematode in several countries [5]. With regard to *A. vasorum*, there is clear evidence of both an increase in the number of cases reported within known endemic foci (e.g. France, Denmark and UK) and the appearance of new foci in several regions previously free of infection [6,9,14,15]. There is great interest in understanding the reasons for the spread of French heartworm and to elucidate the factors, global warming or other(s), that may be involved. The major hindrance for such studies on *A. vasorum *is the lack of detailed information on key biological features of the nematode, e.g. influence of climatic factors on development and transmission in the vectors and the role of the vector species themselves. This information is necessary before computer modelling studies, similar to those used for *D. immitis*, can be utilized. Preliminary work has been carried out using a climatic envelope to address at least the geographic regions at risk of spread and/or invasion by *A. vasorum *[7]. Even though it is based on incomplete data, this climate-matching model showed that there are several European regions, e.g. central-northern areas of Europe and Italy, which offer suitable ecological and epidemiological conditions for the expansion of current endemic foci of *A. vasorum *and the establishment of further new endemic foci. These considerations seem to be supported by the events as reported in Italy. In this country *A. vasorum *infection was first reported over twenty years ago in red foxes [16,17] and for quite some time infection was likely confined to this host until recently, when angiostrongylosis has been reported with increasing frequency in dogs [18-20]. Growing awareness among clinicians and researchers may have played a role in the increase in angiostrongylosis diagnoses in dogs, but the trend in higher incidence observed in other European countries and the concomitant expansion in foxes strongly indicate that the parasite is actually emerging [7,9]. Although lack of awareness and misdiagnosis could have resulted in an underestimation of infection levels in dogs, the high level of pathogenicity associated with *A. vasorum *infection makes it unlikely that clinical cases in previous years were missed. Nonetheless, more studies are necessary to elucidate the potential effects of climatic variables on parasite and intermediate host development rates as well as the transmission patterns in both endemic and non-endemic areas, and to address the question whether global warming and/or other factors actually explain the current spread of *A. vasorum*.

Prediction studies are warranted for two other gastropod-borne lungworms, for which no extensive computer modelling or epidemiological research data are available, i.e. *A. abstrusus *("feline lungworm") and *C. vulpis *("fox lungworm"). Recent reports have indicated a possible expansion in the geographical range of *A. abstrusus *infection and/or a new awareness that infection in cats may be much more common than previously thought [20,21]. In particular there are endemic areas in Europe, like Portugal [22] and Italy [23,24], where the prevalence of this lungworm is ~20% and aelurostrongylosis is a significant cause of feline respiratory disease. The fox lungworm, *C. vulpis*, affects wild and domestic canids in Europe and North America and, though neglected in the past, has recently been detected and/or recognized as a cause of canine respiratory disease in different countries of the Old Continent, e.g. Switzerland, Portugal, Germany [25-27]. Whether the apparent emergence of *A. abstrusus *and *C. vulpis *reflects an increased infection exposure risk or the utilization of better diagnostic methods is unclear. Unlike canine angiostrongylosis, infection of *A. abstrusus *in cats and *C. vulpis *in dogs tends to be insidious even in cases involving clinical disease. Fatal infection of *A. abstrusus *in cats is relatively uncommon and fatal *C. vulpis *infection has never been reported in dogs. Clinicians failing to consider *C. vulpis *infection in cases of dogs suffering signs of chronic cough are most likely to misdiagnose and treat the condition as allergic respiratory disease. Since treatment of allergic respiratory disease is symptomatic, the lungworm infected dog will show clinical improvement and therefore the clinician will have no reason to suspect that they have made a misdiagnosis. The same may be true of cats infected with *A. abstrusus*. Nonetheless, for gastropod-transmitted parasitoses, the geographic spread of slugs and snails driven by conditions of global warming may play a role in the incidence and distribution of these parasites [28-31]. With similar life cycle patterns, the same factors involved in the spread of *A. vasorum *would likely also have an affect on *A. abstrusus *and *C. vulpis*. This remains speculative awaiting further study.

Canine and feline respiratory infection by *E. aerophilus *is considered sporadic and most often sub-clinical. However, in the past decade, clinical cases in animals have been reported [32-35] as well as zoonotic infection in humans [36]. In Europe, the nematode is commonly found in wildlife, but recently it has been identified in companion animals, for instance in both dogs and cats from Italy [20,37] and in dogs in Portugal [26]. Knowledge of epidemiological data (e.g. range of hosts and geographic distribution) of *E. aerophilus *in Europe is fragmentary, thus at the moment it is difficult to assess to what degree this parasite may be spreading or what influence global warming or other factors may have on the current distribution.

Indeed, although climate changes seem to play a key role into the epidemiological variations for pet heartworms and lungworms, other possible factors can not be ruled out. Among them, animal travel, international trade, lack of large-scale surveillance and control programs and, possibly, enhanced awareness of veterinarians and improved diagnostic tools may also play a role [21,38,39]. Thus there is significant risk of introduction of these parasites through the movement of infected animals to previously non-endemic areas as seen with *A. vasorum *[40,41] and *Dirofilaria *spp. [42,43]. In addition, the introduction of competent vectors through commercial trade goods may favour the establishment and spread of parasites to new regions, as already discussed for *Aedes albopictus *and *Dirofilaria *spp. [44,45].

**Figure 1:**
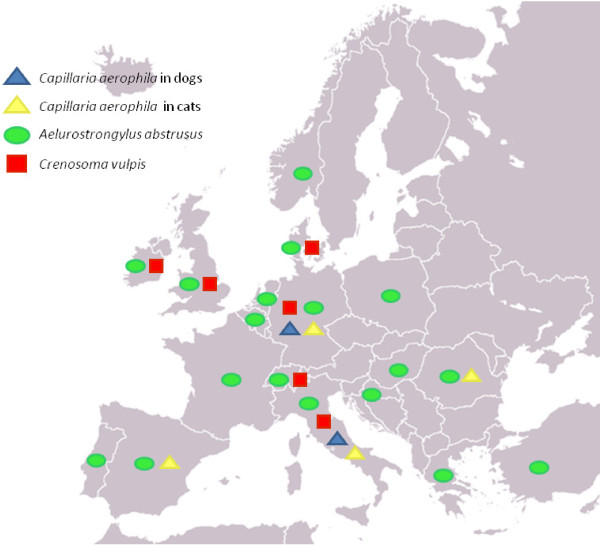
**Reports in Europe of *Eucoleus aerophilus*, *Aelurostrongylus abstrusus *and *Crenosoma vulpis *(Cv)**. C:cats; D:dogs (see also Table 1).

**Figure 2:**
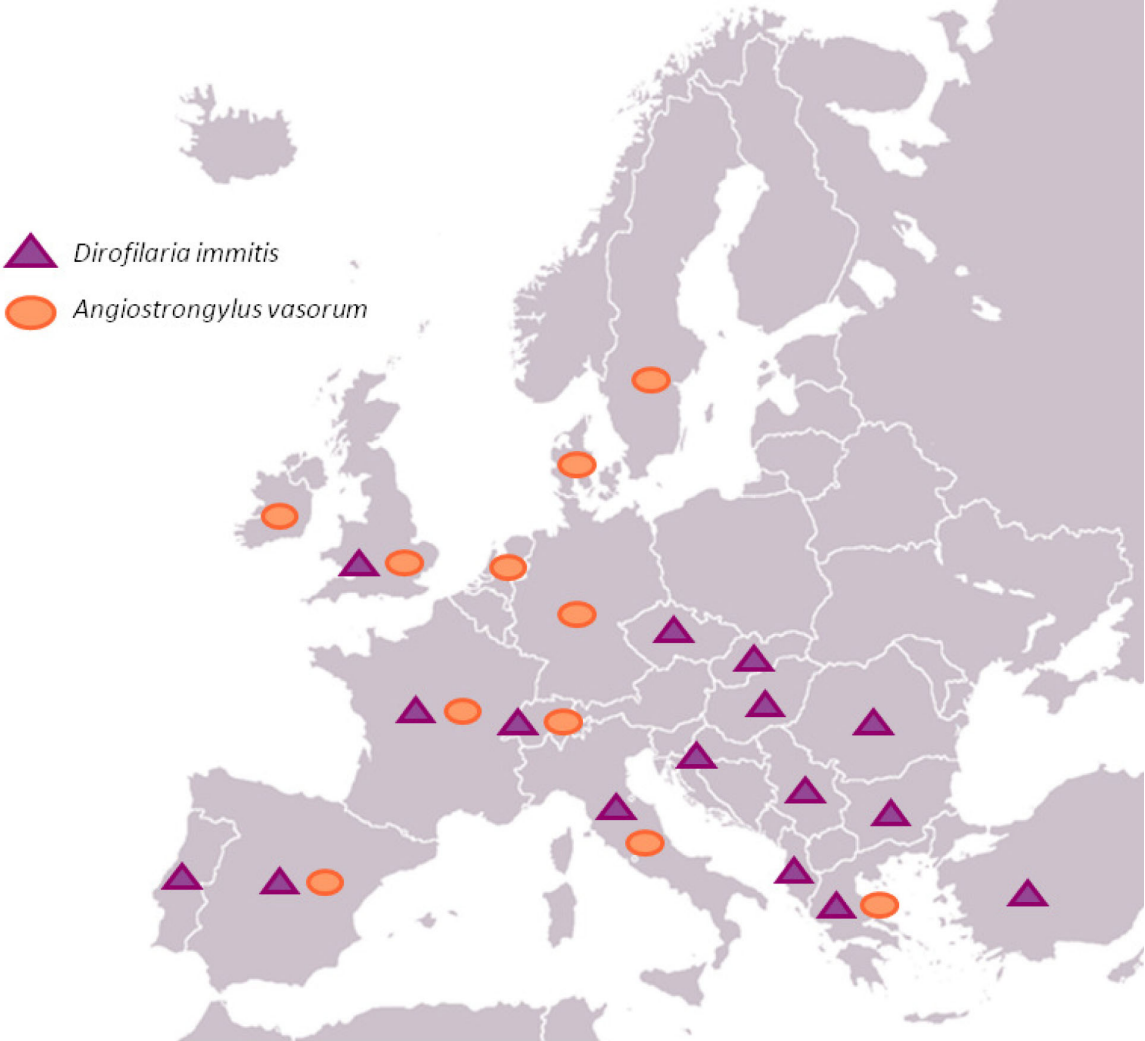
**Reports in Europe of *Dirofilaria immitis *and *Angiostrongylus vasorum *in dogs**. See also Table 2.

Tables 1 and 2 report the prevalence rate or single description/s of cardio-pulmonary nematodes in Europe, while Figures 1 and 2 depict the distribution of lungworms (*C. vulpis*, *A. abstrusus *and *E. aerophilus*) and heartworms (i.e. *D. immitis *and *A. vasorum*).

**Table 1:** Examples of prevalence rates/ranges (%) or single report/s (S) for *Aelurostrongylus abstrusus *(Aa), *Crenosoma vulpis *(Cv) and *Eucoleus aerophilus *(Ea) in some European countries.

Country	*Aa%*	*Cv%*	*Ea% *
Italy	24.4	S	2.8(D)-5.5(C)
France	S	-	-
Switzerland	S	S	-
Spain	1	-	1.3(C)*
Portugal	17.4	-	0.3(D)
Great Britain	S	S	-
Denmark	S	1.4	-
Holland	2.6	-	-
Ireland	S	S	-
Germany	0.7-6.5	0.9-6	0.2(C, D)*
Belgium	S	-	-
Norway	S	-	-
Poland	S	-	-
Hungary	S	-	-
Croatia	0.38-22	-	-
Greece	S	-	-
Romania	5.6	-	3.1(C)
Turkey	S	-	-

C:cats; D:dogs; *The nematode was identified as *Capillaria *spp.

**Table 2:** Examples of prevalence rates/ranges (%) or single report/s (S) for *Angiostrongylus vasorum *(*Av*) and *Dirofilaria immitis *(*Di*) in some European countries.

Country	*Av%*	*Di%*
Italy	S	0.6-80; S(C)
France	S	0.6-6.8
Switzerland	S	1.6
Spain	S	0.6-58.8
Portugal	-	S
Great Britain	S	S
Irland	S	-
Denmark	2.2	-
Netherland	0.8	-
Sweden	S	-
Germany	0.3-7.4	-
Czech Republic	-	S
Slovakia	-	S
Hungary	-	S
Croatia	-	S
Serbia	-	6.2
Romania	-	S
Bulgaria	-	S
Albania	-	S
Greece	1.1	10-34
Turkey	-	S

C:cats; D:dogs.

The information reported in Tables 1 and 2 and Figures 1 and 2 are based on refs [14,15,18-27,37,46-89].

## Pathological significance

Clinical aspects of canine and feline heartworm and lungworm infections have been extensively reported and interested readers are referred to various reviews[9,21,90-94]. Therefore, this chapter is intended to summarize the main aspects of the pathogenic role of cardiopulmonary nematodes in clinical settings and to emphasize their potential role as a zoonotic threat.

These infections present varying clinical outcomes depending on different variables such as worm burden, age and immune response of the infected animal, and concomitant diseases. Given the similar localization of adult stages (i.e. right side of the heart and the pulmonary arteries), angiostrongylosis and cardiopulmonary filariosis may present similar clinical pictures. Infection in dogs with either parasite can result in cardiac damage progressing to right-sided congestive heart failure and signs of exercise intolerance, ascites, tachycardia, severe dyspnoea and syncope. Respiratory signs such as cough, dyspnoea and tachypnoea can also occur with either heartworm. Cardiopulmonary filariosis in cats can involve a prolonged asymptomatic prodromal period ending with the sudden death of the animal. A distinctive clinical sign for *A. vasorum *infection is the presence of bleeding disorders and coagulopathies, likely due to disseminated intravascular coagulation [9,94,95]. Such a disorder triggers a wide range of symptoms which may have serious consequences [94], for instance neurological signs due to bleeding into the brain or spinal cord [96] or massive bleeding in body cavities [19].

Symptoms in cats infected by *A. abstrusus *are not always detectable, as the disease may be asymptomatic or subclinical [97-99]. The mild forms, which are frequent in adult animals and/or in the case of low worm burdens, may be self-limiting and respiratory symptoms gradually and spontaneously disappear within weeks [98]. When present, the clinical signs are due to the inflammatory response caused by the eggs shed by the adult females and the migration of the first stage larvae (L1) up the bronchial tree causing lesions in the pulmonary alveoli, bronchioles and local arteries [100]. Respiratory signs, e.g. mild to intense cough, sneezing, mucopurulent nasal discharge, dyspnoea, open-mouthed abdominal breathing, and even death, are most often observed in young, debilitated and/or immunosuppressed animals [48,101-103].

Similar clinical signs are present in animals infected by *E. aerophilus *(cats and dogs) and *C. vulpis *(dogs). In general, pulmonary capillariosis is typically characterized by a chronic bronchitis and infected animals may present with minimal respiratory signs (e.g. bronchovesicular sounds) to inflammation, sneezing, wheezing, and chronic dry cough. When bacterial complications intervene, cough may become moist and productive and bronchopneumonia and respiratory failure occur. Heavy parasite burdens may lead to mortality [35,104-106]. Canine crenosomosis is usually characterized by bronchitis with a dry, non-productive cough that can be elicited by tracheal palpation and may be accompanied by gagging [107,108]. High parasitic burdens may, in turn, induce mucoid or mucopurulent discharge in the airways and the cough is chronic and productive. Although cough may be severe, fatal infections in dogs have never been reported [93]. In most cases, mild to moderate bronchial patterns that may have a diffuse interstitial component most evident in the diaphragmatic lobes are observed on radiographs [25,62,107-112].

While the metastrongyloid infections are restricted to their animal hosts, *D. immitis *and *E. aerophilus *also have zoonotic potential. Indeed, pulmonary capillariosis in humans is an occasional event, with about a dozen cases described to date in the scientific literature from Asia, Africa and Europe [36,113-115]. Human infection is characterized by bronchitis, coughing, mucoid sputum, presence of blood in the mucous, fever, and dyspnoea. The last described report was a cryptic case of infection in a woman from Serbia that resembled a bronchial carcinoma [36]. The infected patient had a productive cough with purulent expectorates and the computed tomography scan revealed bronchopneumonic shadows, and spotted, partially confluent, nodular, and banding infiltrative lesions of the pulmonary parenchyma, similar to those occurring in lung cancers. More in depth analysis revealed the presence of *E. aerophilus *eggs in the bronchial biopsy, thus suggesting that the parasites likely died in the bronchial tree, causing abscesses which appeared as tumor-like lesions. As illustrated by this case, the diagnosis of human capillariosis may be a serious challenge due to the likelihood of infection being mistaken for cancer [36]. Although the subcutaneous dwelling *Dirofilaria repens *is the most common filarial zoonotic pathogen in Europe, the possibility that human infections by *D. immitis *may occur should be taken into serious account. Human infection with *D. immitis *has been mostly reported from USA and Far East especially where the parasite is endemic in the dog population [92,116]. Nonetheless, due to the frequency of subclinical infections and the lack of specific diagnostic tests, the actual incidence and prevalence of human cases is likely underestimated worldwide [116,117] and this could be also true for European regions where the infection is endemic (e.g. North of Italy). In general, after the bite of an infected mosquito, the vast majority of larvae are eliminated in the human host by the immune system [118]. When some larvae continue their migration, thus reaching the pulmonary arteries and their branches, they elicit a local inflammatory response which can kill the larvae [117]. Thereby, the worms are incorporated into granulomas, appearing as typical 'coin'-like densities on radiographic examination. The majority of these infections are asymptomatic but in some cases infected people may present with pneumonitis-like symptoms [117,119-121]. As is the case with *E. aerophilus*, cardiopulmonary specialists may be faced with significant diagnostic challenges, given the lack of adequate diagnostic tests and the number of other disease conditions (e.g. fungal, bacterial and neoplastic diseases) which can result in similar pulmonary lesions. In addition, infection involving extra-pulmonary tissues have also been observed, e.g. in a branch of the left testicular artery, within a hepatic nodule in the peritoneal cavity and the eye [116,117,122].

Even though most of the past cases of human infection in Europe have been found to be caused by *D. repens*, further studies are warranted to elucidate the current rate of human infections by *D. immitis *in the Old Continent. This is particularly true considering the increasing distribution of the parasite in different geographical areas of Europe and the spreading of new competent vectors, which indeed pose a present and future public hazard [5].

## Are heartworms and lungworms an actual diagnostic challenge for parasitologists and practitioners?

Cardiopulmonary parasitoses in dogs and cats cannot be easily diagnosed clinically, because several other conditions should be considered in the differential diagnoses as well as sub-clinical or atypical infections that may occur. Infections caused by *A. vasorum *and *D. immitis *are characterized by a wide spectrum of clinical features, ranging from a subclinical or mild disease to a severe, potentially fatal manifestation. Parasitoses caused by *A. abstrusus*, *C. vulpis *and *E. aerophilus *are also difficult to interpret in current clinical practice since the signs of disease are similar or the same as those associated with a series of other respiratory conditions. In general, clinical signs such as fatigue, cough, sneezing, wheezing, mucopurulent nasal discharge, dyspnoea, open-mouthed abdominal breathing and bronchopneumonia, oblige veterinarians to include a multiplicity of diseases and distresses in the differential diagnosis, e.g. viral, bacterial and fungal diseases, non-infective inflammatory (e.g. nasopharyngeal polyps and allergic bronchitis) disorders, foreign bodies and respiratory neoplasms [21,33,34,123]. Additionally, these infections often present nonspecific abnormal radiographic and haematological findings, thus proper diagnostic methods are critical to achieve a timely and reliable diagnosis for cardiopulmonary nematodes affecting cats and dogs [9,21,33,34,123-125].

**Figure 3:**
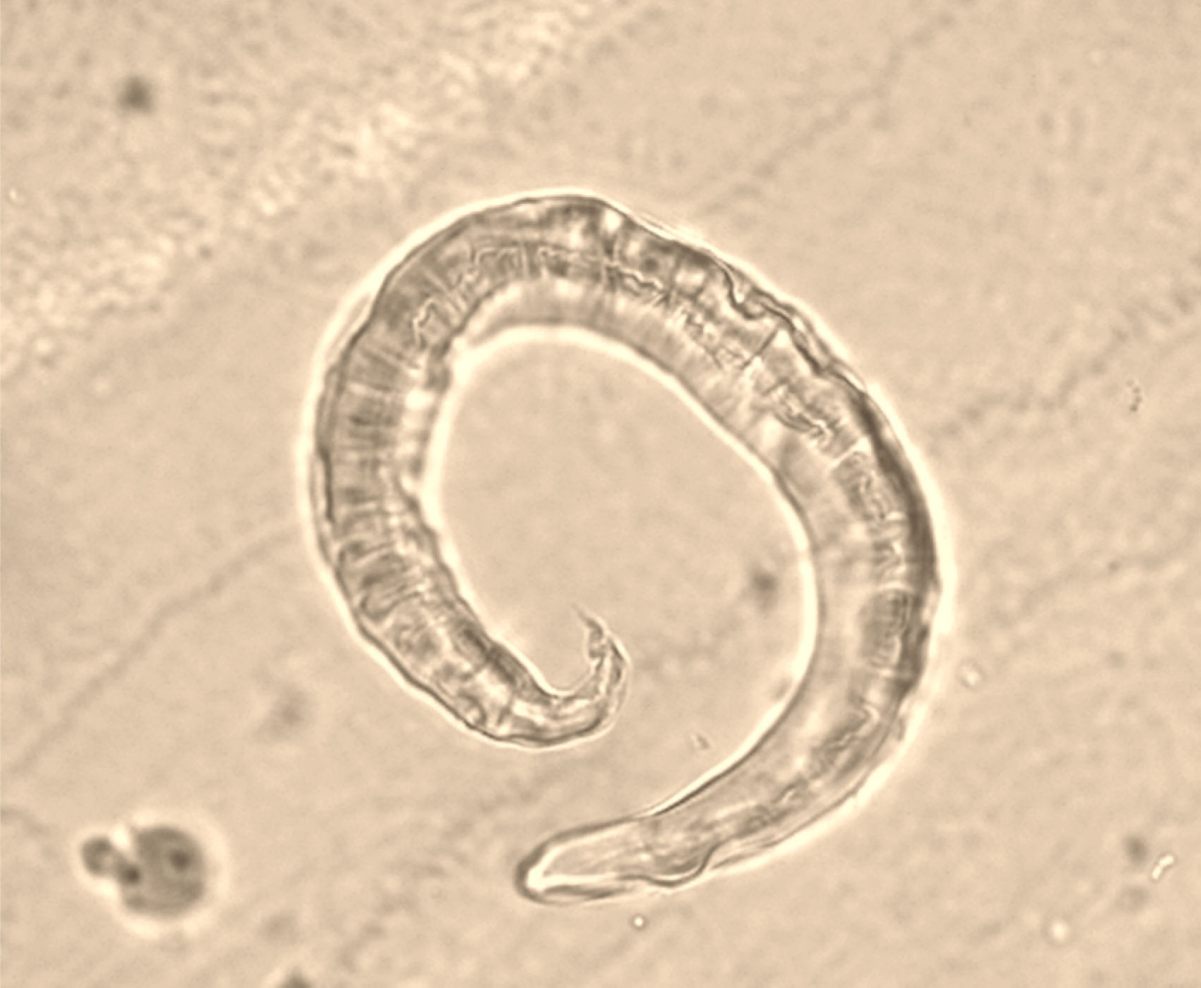
**Floatation with Zinc Sulphate: *Angiostrongylus vasorum*, dehydrated first stage larva**.

**Figure 4:**
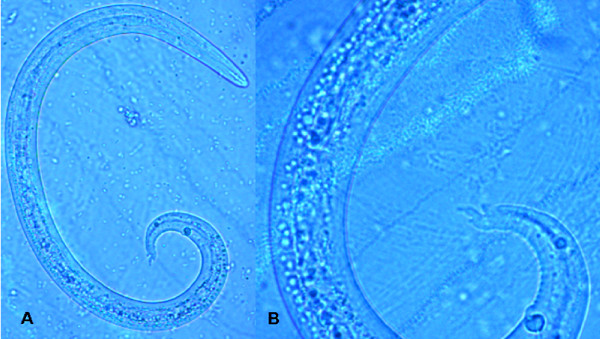
**Baermann test: *Aelurostrongylus abstrusus*, first stage larva (A) and magnification (B) of the S-shaped caudal end with the typical dorsal spine**.

**Figure 5:**
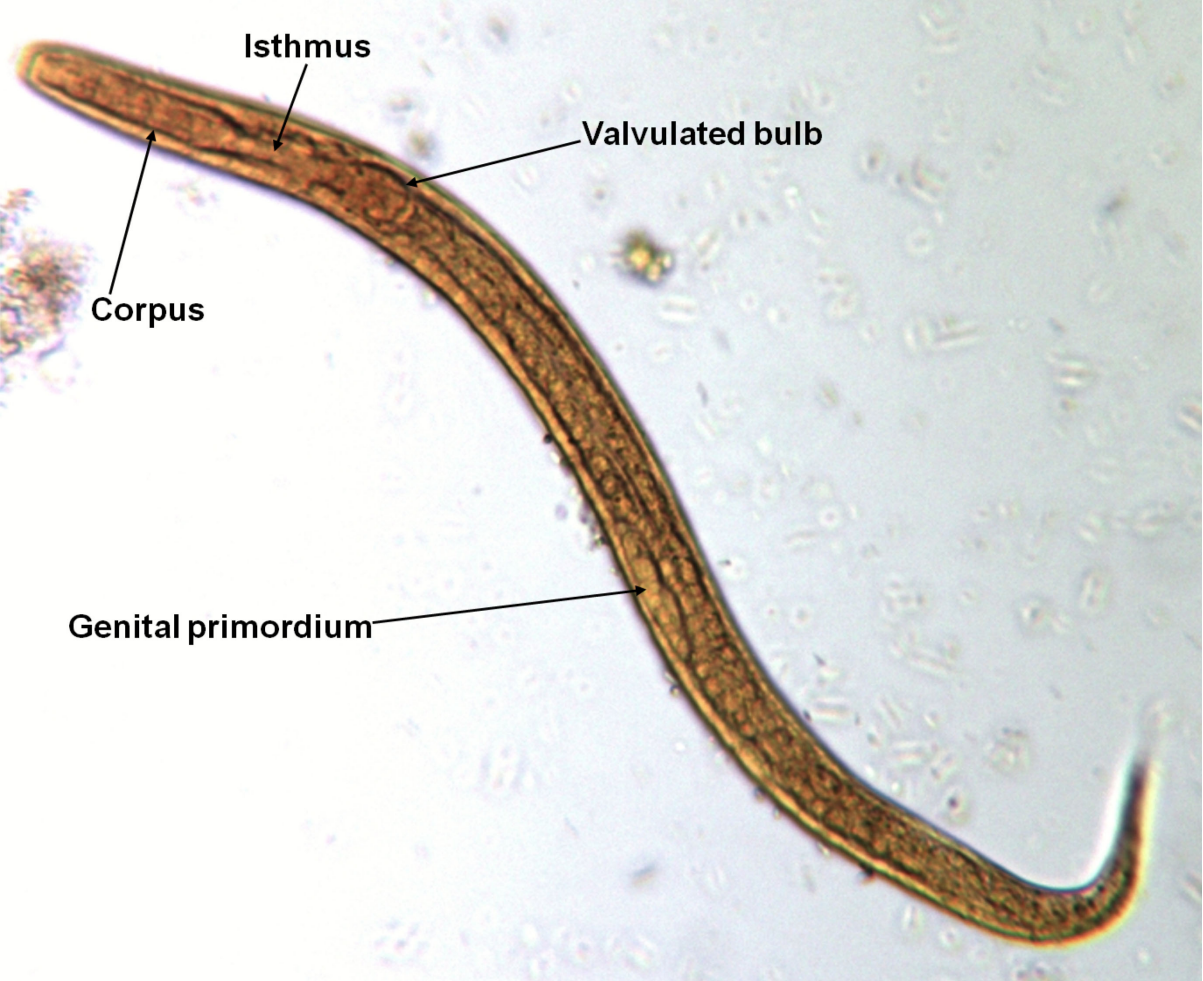
**Baermann test: *Strongyloides stercoralis*, first stage larva, iodine stained**.

**Figure 6:**
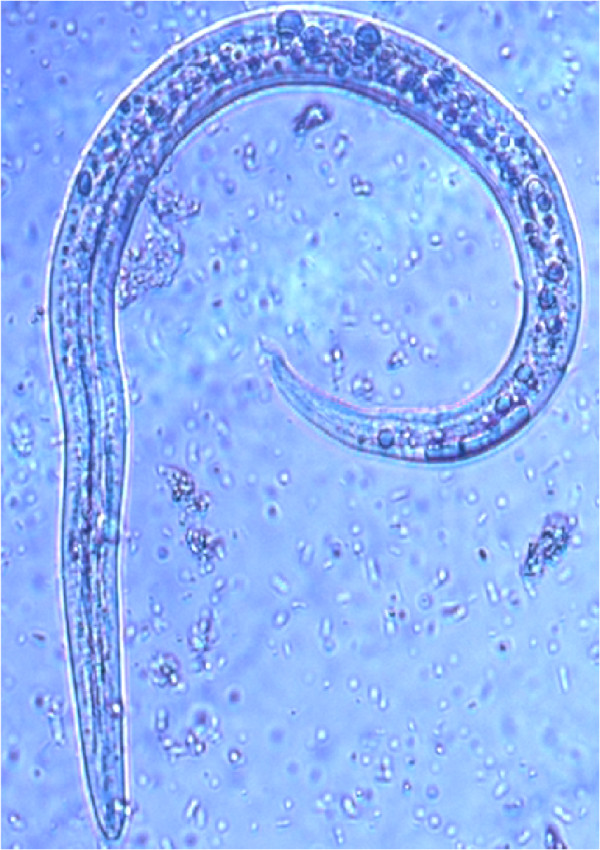
**Baermann test: *Angiostrongylus vasorum*, first stage larva**.

**Figure 7:**
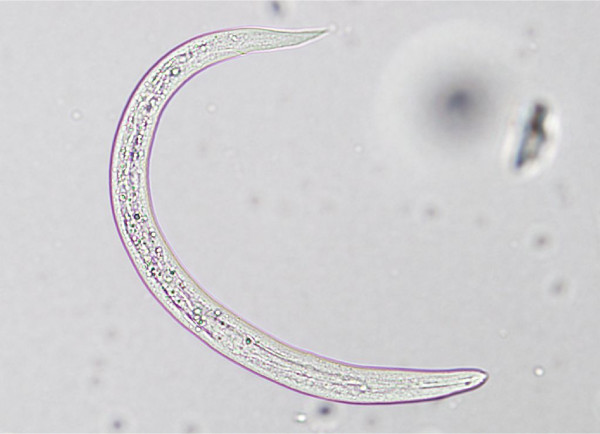
**Baermann test: *Crenosoma vulpis*, first stage larva**.

The accurate and definitive diagnosis of lungworm infections is achieved by the identification of parasitic biological stage(s) in a series of samples. For nematodes whose eggs hatch in the bronchial tree of the infected animals, i.e. *A. vasorum*, *C. vulpis *and *A. abstrusus*, L1 s are the "target" to be identified in the faeces by using copromicroscopic techniques such as direct smears, faecal flotation and Baermann examinations. Direct smears are inexpensive and easy to perform but have poor sensitivity due inadequate sample size and will only detect infections in animals that are shedding large numbers of larvae in the faeces [21,126]. Faecal flotation examinations also lack detection sensitivity due to issues of sample size and the damaging effects of the flotation media on the larvae (Figure 3). In fact, high specific gravity concentrated salt or sugar solutions tend to induce larval osmotic damage due to dehydration. Larvae may be completely damaged and therefore undetectable or suffer a loss of morphologic detail rendering accurate larval identification difficult to impossible, especially for inexperienced diagnosticians [21]. Zinc sulphate (specific gravity = 1.18 - 1.2) appears to be the most reliable media for use in the detection of L1 s on fecal flotation but still may miss 40-90% of the positive animals [127]. The Baermann faecal examination method remains the gold standard for the diagnosis of the infections caused by *A. vasorum*, *C. vulpis *and *A. abstrusus*, due to the positive hydro-/thermo-tropism exhibited by live L1 [21,93]. When the larvae are present in the stool samples, a thorough morphometric and morphological examination is required for an accurate identification of L1. Lungworm larvae should be differentiated from i) each other, ii) those of other less common parasites, iii) hookworm larvae that may be present in samples that have been allowed to incubate and iv) free-living or plant parasitic nematodes that may be present in samples collected from the ground. In addition to *A. abstrusus *(Figure 4), another less common parasitic larvae that may be detected using the Baermann technique in feline fecal samples includes the lungworm *Oslerus rostratus*. In canine samples, larvae of the lungworms *Filaroides *spp. and *Oslerus osleri *as well as those of the intestinal parasite *Strongyloides stercoralis *(Figure 5) may also be detected and need to be differentiated from *A. vasorum *(Figure 6) and *C. vulpis *(Figure 7) [93]. The finding of L1 s of *Filaroides *spp. and *Oslerus *spp. in samples examined using the Baermann method is unlikely because larvae of these nematodes are lethargic and therefore do not migrate out of the faeces. Although false negative results commonly occur, detection of these larvae in faeces is more reliably accomplished through centrifugal faecal flotation using zinc sulfate solution (specific gravity = 1.2) than with the Baermann technique. Table 3 details the key morphometric and morphological characters (i.e. length, width, esophageal and tail morphology) to be evaluated when nematode larvae are found in canine or feline faeces. The presence of nematode contaminants in the faeces greatly complicates the evaluation of the sample. Due to the large number of species of free-living/plant parasitic nematodes involved, there is no single morphological character that allows the diagnostician to differentiate the contaminants from the parasitic L1. A diagnostician should suspect the possibility of contaminants if the sample contains adult stages (i.e. males with spicules, females with vaginal opening and uterus containing eggs) or the size measurements of the nematodes recovered are inconsistent with the range expected for parasitic L1 (i.e. 150 - 412 microns). Since *S. stercoralis *may develop a free-living generation if the faecal sample is allowed to incubate, even the presence of adult stages does not definitively indicate contamination has occurred. When contamination has been suspected, specific instructions should be given to the animal's owner on proper sample collection (faeces should be collected immediately after deposit on the ground) and the animal should be re-tested.

**Table 3:** Cardiopulmonary nematodes affecting dogs and cats: differential features of first stage larvae found in faecal samples.

Nematode	Length (μm)	Oesophagus	Caudal end	Refs
*Aelurostrongylus abstrusus*	300-390	Non-rhabditiform; 1/3 - 1/2 the length of the larvae	Notched and S-shaped	[97,131]
				
*Angiostrongylus vasorum*	310-400	Non-rhabditiform; 1/3 - 1/2 the length of the larvae	Tip with a dorsal spine and a sinus wave curve	[21,93]
				
*Oslerus rostratus*	335-412	Non-rhabditiform; 1/3 - 1/2 the length of the larvae	Constriction anterior to the end; tip with a kinked appearance	[21,93]
				
*Oslerus osleri* *Filaroides *spp.	~250	Non-rhabditiform; 1/3 - 1/2 the length of the larvae	Absence of dorsal spines and S-shaped end with a slight kink	[91,93]
				
*Crenosoma vulpis*	240-310	Non-rhabditiform; 1/3 - 1/2 the length of the larvae	Pointed and straight tail	[127]
				
*Strongyloides stercoralis*	150-390	Rhabditiform (corpus, isthmus, valvulated bulb); 1/4 the length of the larvae	Pointed and straight tail	[93]
Hookworm larvae	290-360			

Although the Baermann examination is the gold standard for diagnosing angiostrongylosis, aelurostrongylosis and crenosomosis, this method has inherent limitations. It is relatively time-consuming (i.e. 12-48 hrs) and false negative results can occur due to prepatent infections and the intermittent fecal larval shedding pattern typical of metastrongyloids. Thus, repeated examinations (e.g. samples collected for three consecutive days) are necessary to increase the sensitivity [21,93].

Molecular studies have overcome the inherent limitation of conventional methods for the diagnosis of aelurostrongylosis and angiostrongylosis. The DNA of the feline lungworm is detectable by a nested PCR based on genetic markers within the ribosomal DNA. This DNA-based assay may be applied to both faecal and pharyngeal swab samples with a specificity and sensitivity of about 100% and 97% respectively, which are values much higher than those that the classical methods may achieve [128]. Very recently, innovative techniques have been assessed for the French heartworm as well. A real-time PCR specific for *A. vasorum *ribosomal regions was developed to amplify parasite DNA from both definitive and intermediate host samples. For clinical purposes, this assay was able to detect a single L1 in 200 μl of blood and in 200 mg of faeces. This method showed a more efficient means for the diagnosis of canine angiostrongylosis with a lower limit of detection than the Baermann method [129]. Additionally, circulating adult worm antigen has been detected in the serum of *A. vasorum *infected dogs through the use of a sandwich-ELISA. A sensitivity of 92% and a specificity of 100% were reported for the sandwich-ELISA [130]. Detection of circulating adult worm antigen has great promise as a method for the diagnosis of angiostrongylosis in dogs.

**Figure 8:**
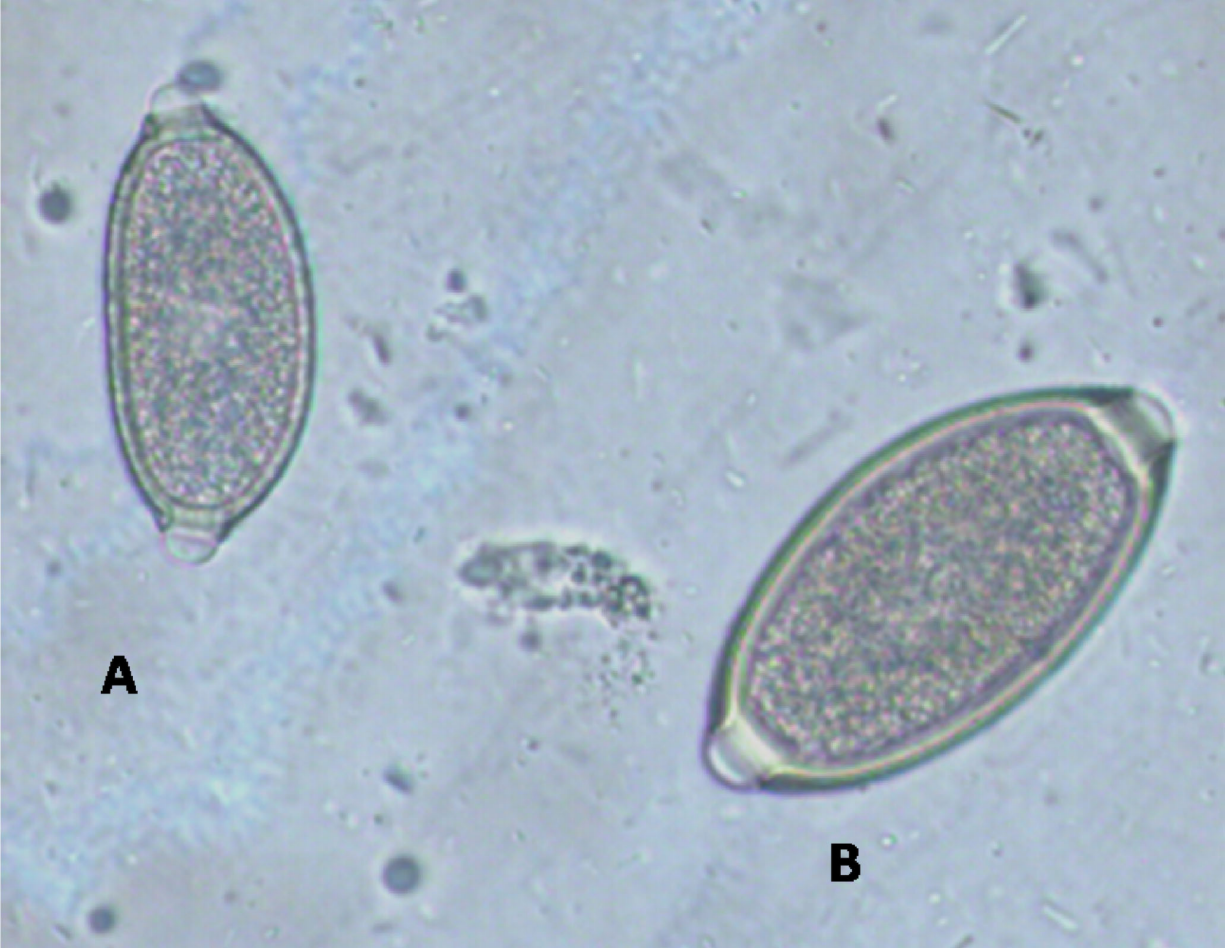
**Floatation with Zinc Sulphate: eggs of *Eucoleus aerophilus *(A) and *Trichuris vulpis *(B)**.

**Figure 9:**
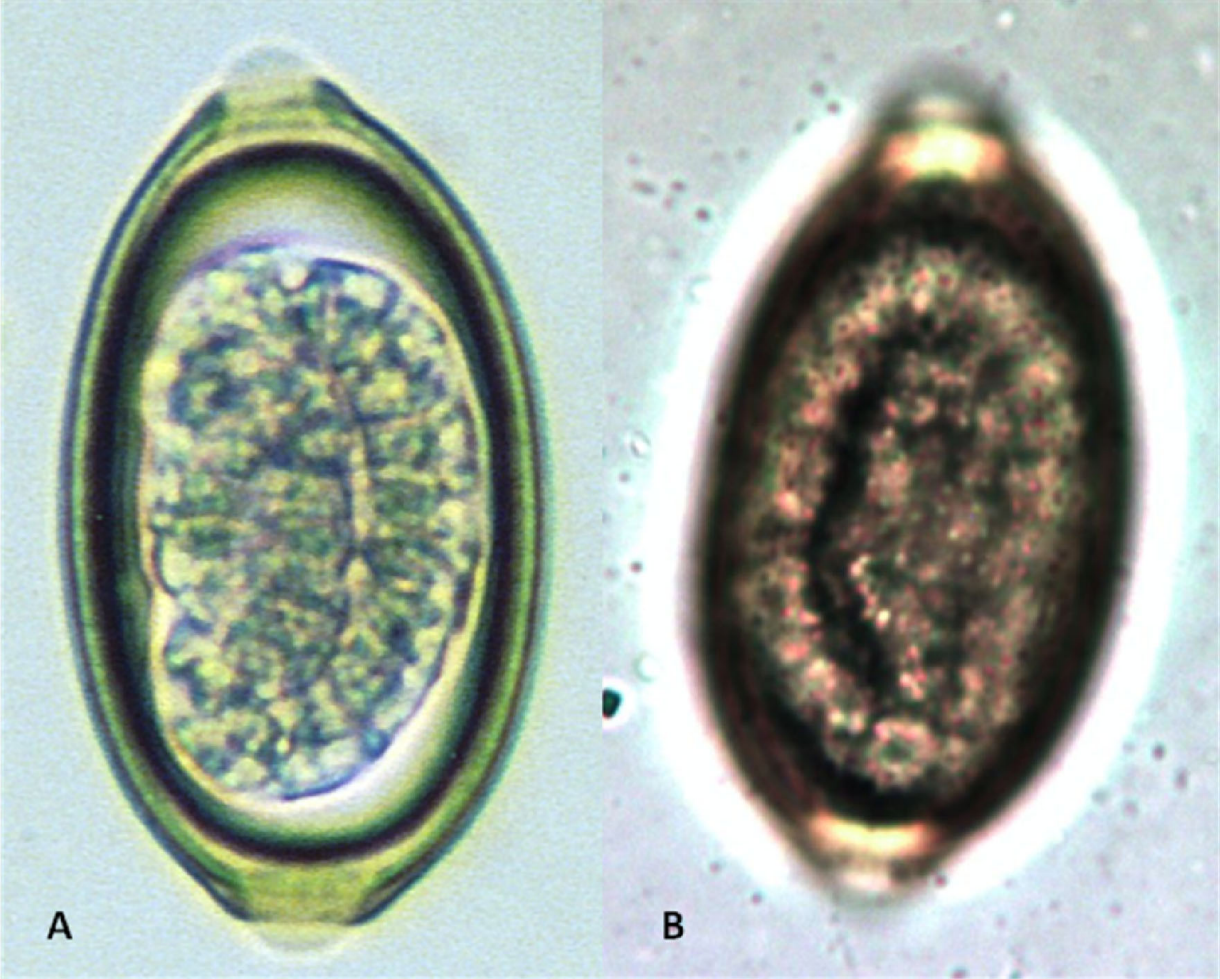
**Floatation with Zinc Sulphate: eggs of *Eucoleus boehmi *(A) showing the pitted wall surface (B)**.

Canine and feline capillariosis is diagnosed by the detection through standard fecal flotation of the typical trichuroid eggs passed in the faeces by the infected hosts. The eggs of *E. aerophilus *(Figure 8A) need to be differentiated (Table 4) from the eggs of intestinal whipworm *Trichuris *spp. (Figure 8B), the nasal capillarid *Eucoleus boehmi *(Figure 9A) and those of the stomach capillarid *Aonchotheca putorii*. Indeed, the similarity in morphological features of *Eucoleus *and *Trichuris *eggs make accurate identification a challenge for microscopists. The possibility of misdiagnosis and the practice of identifying eggs only as "capillarid" or "trichuroid" have likely complicated the interpretation of field survey data with respect to prevalence of infection for the various capillarids [37,93], which is likely in mixed infections (Figure 8). When the barrel-shaped eggs are found in samples following copromicroscopic analysis with flotation solutions (specific gravity = 1.2-1.35) their shell wall surface pattern (Figure 9B, Table 4), size and plug morphology must be carefully examined (Table 4) to achieve a reliable diagnostic result. At the moment, no molecular methods have been developed for the diagnosis of the infections caused by *E. aerophilus *and *C. vulpis*, although genetic investigations are presently ongoing to characterize ribosomal and mitochondrial diagnostic markers (Traversa et al., unpublished).

**Table 4:** Capillariid and trichuroid nematodes affecting dogs and cats: differential features of eggs found in faecal samples.

Species	Size	Morphological features	Refs
*Eucoleus aerophilus*	60-85 μm long 25-40 μm wide	Bipolar plugs asymmetrical, outer shell densely striated and with presence of a network of anastomosing ridges	[37,93,105]
*Eucoleus boehmi*	50-60 μm long 30-35 μm wide	Tiny pits on the surface of the egg wall	[37,90,93]
*Trichuris *spp.	70-80 μm long 30-50 μm wide	Symmetrical, presence of ringed thickening at the base of the bipolar plugs, smooth egg wall	[93,131]
*Aoncotheca putorii*	56-72 μm long 23-32 μm wide	Bipolar plugs asymmetrical, sides of shell wall nearly parallel, longitudinal network of anastomosing ridges on shell wall surface	[37,93]

**Figure 10:**
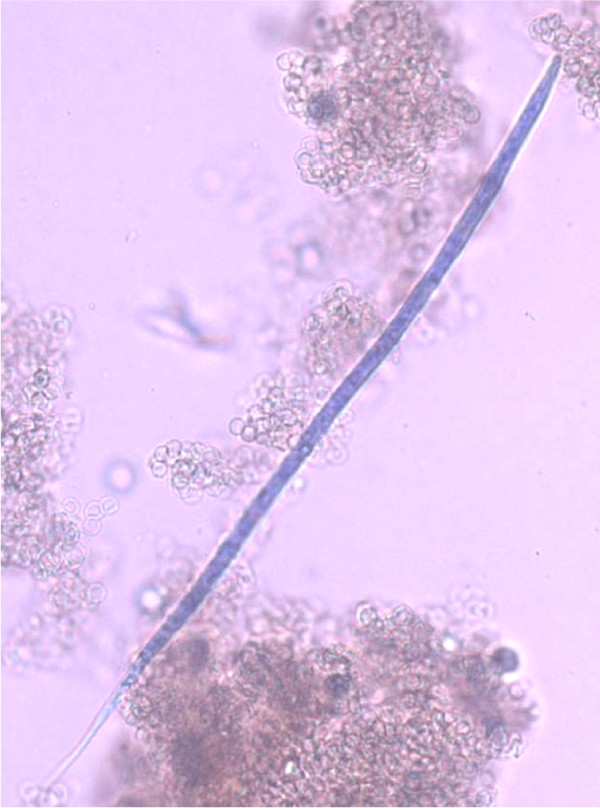
**Knott's method: *Dirofilaria immitis *microfilaria**.

**Figure 11:**
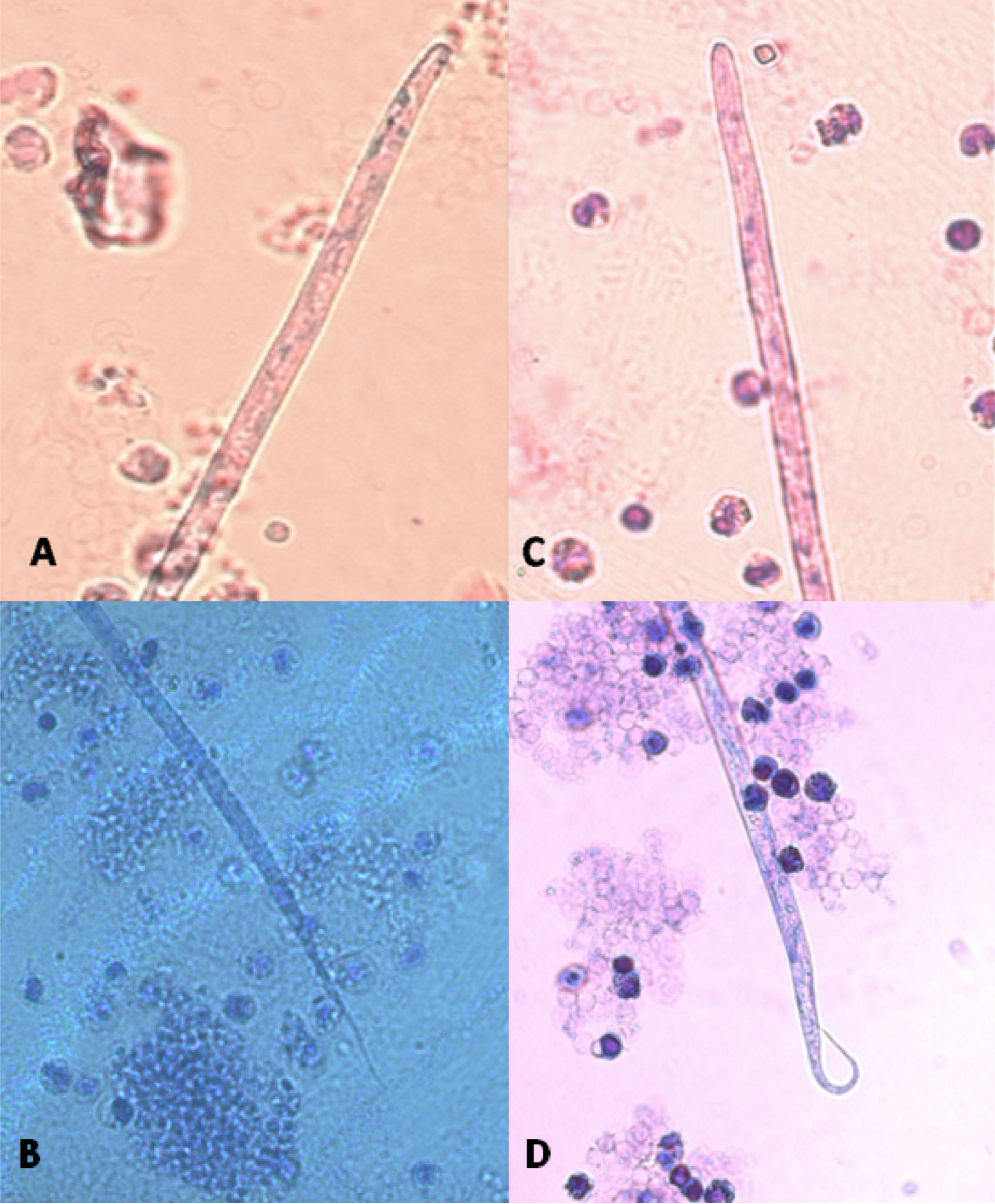
**Knott's method: Anterior and posterior end of *Dirofilaria immitis *(A-B) and *Dirofilaria repens *(C-D)**.

**Figure 12:**
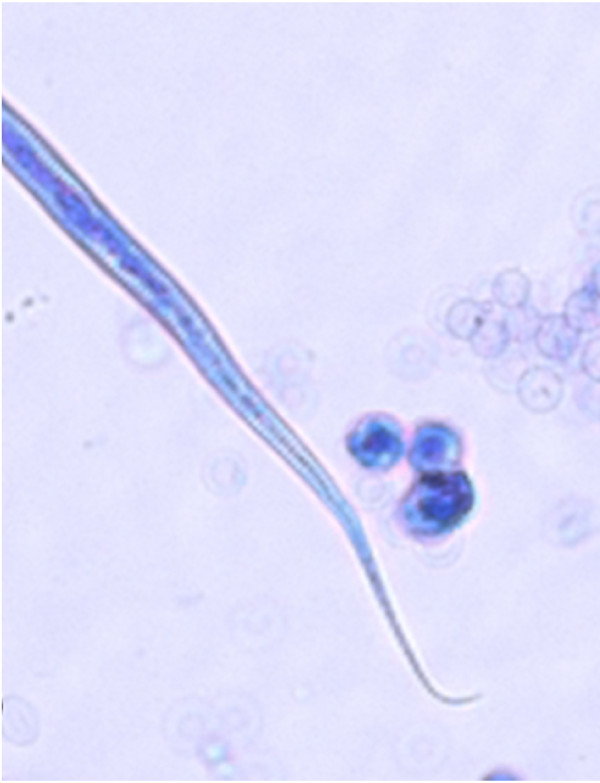
**Knott's method: Posterior end of *Acanthocheilonema *(*= Dipetalonema*) *reconditum***.

In contrast to most of the other cardiopulmonary nematodes, blood is the biological sample to examine for the diagnosis of heartworm infection. Adult female *Dirofilaria *produce live microfilariae (i.e. L1s) that can be detected in the bloodstream of infected animals by several microscopic techniques, e.g. the Knott's method [92]. Microfilariae of *D. immitis *(Figure 10) must be differentiated [106,131] from those of other (non cardiopulmonary) filarioids infecting dogs and cats including *D. repens *and *Acanthocheilonema *(*= Dipetalonema*) *reconditum *based on differences in morphology and size measurements (Figures 11, 12, Table 5). Anterior-end morphology of *D. immitis *(gently tapered) differs from that of *A. reconditum *and *D. repens *(blunt). An artifact of fixation in 2% formalin in the Knott's method can cause a distinctive curve in the tail of *A. reconditum *(ie "button-hook" tail) and *D. repens *while the tail of *D. immitis *remains straight. However, the occurrence of the tail artifact is highly variable and therefore, *A. reconditum *and *D. repens *cannot be ruled out as possibilities in microfilaria with a straight tail. There are definite differences in size between the microfilaria of these three species (see Table 5) that can be used to accurately identify the parasite involved.

**Table 5:** Major filarioid nematodes affecting dogs and cats: differential features (derived from Refs 106 and 131) of microfilariae found in blood samples by Knott's examination.

Species	Length (μm)	Width (μm)	Posterior end	Anterior end
*Dirofilaria immitis*	260-340	5-7.5	Straight and thin	Gently tapered
*Dirofilaria repens*	325-380	5-8	Umbrella-like	Blunt
*Acanthocheilonema reconditum*	240-290	4.5-5.5	Button-hooked	Blunt, with a prominent cephalic hook

Microfilaria detection tests lack sensitivity in that up to 20-30% of *D. immitis *infected dogs are not microfilaremic due to single-sex infections, low worm burdens, immune reactions or past administration of parasiticides with microfilaricidal activity [92]. A much more reliable method for diagnosis of *D. immitis *infection in dogs is through the use of commercial kits for the detection of circulating adult female antigen in the blood. Nonetheless, there is a small proportion of microfilariemic animals which may have negative results using the antigen detection tests, due to such reasons as low worm burden or possible persistence of microfilariae after the death of adult worms [87,92].

It is also worthy of note that the detection of circulating larvae in cats is infrequent due to low and/or short-lasting periods of microfilaremia and non-patent infections [92]. In cats the *D. immitis *antigen detection kits may detect infections caused by a low number of female adults but in these animals, these assays have inherent limitations due to the great possibility of male worm only infections, which can be fatal even in the presence of a single nematode. In general in cats a negative result using antigen detection kits can not rule out the possibility that animals are infected with only male worms, a single female filaria, non-mature worms or old adult worms [92].

To overcome the limits of classical methodologies, several recent studies have been undertaken to differentiate between the various filarioid parasites of pets irrespective of their life cycle stage through the use of molecular techniques. Indeed, *D. immitis*, *D. repens *and *A. reconditum *may be differentiated using interspecific variability in their ribosomal or mitochondrial DNA by RFLP, PCR amplifications with species-specific primers or with universal primers yielding species-specific amplicons [132,133]. The usefulness of molecular tools for identifying *D. immitis *has been recently demonstrated in epidemiological and clinical studies [87,134,135].

## Treatment and control: what's new?

Due to a perception of lack of importance (with the exception of *D. immitis*), there has been very little study until the last decade with respect to anthelmintic efficacies for the treatment of the cardiopulmonary parasites in dogs and cats. Recommended treatment protocols for the most part have been anecdotal and empirically derived.

Even though several therapeutic options have been investigated for the treatment of aelurostrongylosis and angiostrongylosis, benzimidazoles and macrocyclic lactones have been widely used in different formulations to treat these infections. In suspension or in tablet form, fenbendazole, administered daily for 5 days at 20 mg/kg or 15 days at 50 mg/kg was effective in the treatment of feline aelurostrongylosis [136,137]. Administration of fenbendazole at a dosage of 50 mg/kg *per os *daily for 3 consecutive days has been also reported to be effective in the treatment of cats infected with *A. abstrusus *[138,139]. An 18.75% fenbendazole oral paste has become licensed for the treatment of *A. abstrusus *infection in cats at this dosage in the UK. Other anthelmintics have also been tested against *A. abstrusus*, with disappointing results involving controversial or inconsistent efficacies, issues of palatability and inconvenient dosing schedules, toxicosis, side effects and *off label *dosages [97,140,141]. For instance, ivermectin has been used *off label *to treat cats infected with *A. abstrusus*, however, limited activity and/or the need for subsequent administrations [137,142,143] and possible life-threatening side effects in young animals [144] discourage its use as a treatment for this parasitic infection. Recently, the efficacy and safety of the UK formulation of fenbendazole (18.75%) oral paste has been compared with those of two spot-on formulations, one containing the neonicotinoid ectoparasiticide imidacloprid 10% and the macrolactone endo-ectoparasiticide moxidectin 1%, and the other contained the anthelmintic octadepsipeptide emodepside 2.1% and the cestodicide/trematodicide praziquantel 8.6% [145,146]. The three formulations were found to be safe and effective in the treatment of aelurostrongylosis in naturally infected cats. The emodepside 2.1%/praziquantel 8.6% spot-on and the fenbendazole paste showed similar activities while the imidacloprid 10%/moxidectin 1% spot-on was superior in efficacy compared to the other two preparations [145,146].

An analogous spot-on formulation containing imidacloprid 10%/moxidectin 2.5% was shown to be effective for both therapy and prevention of canine angiostrongylosis. A single application of this formulation demonstrated an efficacy (85.2%) similar to that observed in dogs treated with fenbendazole (91.3%) daily at 25 mg/kg for 20 days [147], and also equal to that achieved previously (84.8%) using milbemycin oxime (0.5 mg/kg) given orally once a week for 4 weeks [148]. The same imidacloprid 10%/moxidectin 2.5% spot-on formulation has been evaluated for its effective and safe larvicidal activity in dogs experimentally infected with *A. vasorum *[149]. A single topical application was 100% effective in the elimination of fourth stage larvae (L4) and pre-adults of the parasite, thus preventing patent infections. Use of this topical anthelmintic would likely prevent or, at the least, minimize the potential for severe cardiopulmonary tissue damage in dogs due to *A. vasorum *infection. On this basis, a monthly anthelmintic administration with this formulation would be a powerful tool for preventing the establishment of adult stages of *A. vasorum *and the clinical onset of the disease, especially in endemic areas [149]. Another option for prevention of canine angiostrongylosis is milbemycin oxime. An efficacy of 85% was reported in dogs given milbemycin oxime (0.5 mg/kg) at 30 and 60 days after experimental infection with *A. vasorum *[148].

Various anthelmintics have been used to treat dogs infected with *C. vulpis*. A single topical application of the imidacloprid 10%/moxidectin 2.5% spot-on was found to have an efficacy of 100% in dogs experimentally infected with *C. vulpis *[150]. A single oral dose of milbemycin oxime (0.5 mg/kg) had an efficacy of 98-99% when used in the treatment of dogs experimentally infected with *C. vulpis *[151]. Other treatment options for which efficacy data is unknown but have been reported to be effective when used in cases of natural *C. vulpis *infection in dogs include fenbendazole (25-50 mg/kg, oral, daily for 3-14 days) and febantel (14 mg/kg, oral, daily for 7 days) [108,110,152].

In summary, options that have been used successfully to treat dogs infected with *A. vasorum *or *C. vulpis *include oral fenbendazole, febantel and milbemycin oxime and topical moxidectin-imidacloprid. Oral fenbendazole and topical moxidectin/imidacloprid and emodepside/praziquantel have been used to treat cats infected with *A. abstrusus*. In addition to efficacy, choice of anthelmintic may be influenced by such issues as ease of administration and whether a single or multiple treatments are required. Administration of oral anthelmintics may be difficult in fractious animals (especially cats) or animals that are depressed or moribund.

The least and most investigated of the cardiopulmonary nematodes concerning anthelmintic treatment are *E. aerophilus *and *D. immitis*, respectively. Little information has been published on anthelmintic treatment of capillariosis. Oral fenbendazole (50 mg/kg, daily for 14 days) was reported to be effective in the treatment of one dog infected with *E. aerophilus *[35]. Subcutaneous injection of abamectin (0.3 mg/kg, repeated in 14 days) appeared to be effective in the treatment of a cat infected with *E. aerophilus *[32]. Given the lack of published reports on the treatment of feline and canine *E. aerophilus *infection, further studies are warranted to evaluate the efficacy and safety of anthelmintics against this lungworm.

With regard to *D. immitis*, the arsenical melarsomine dihydrochloride is the most commonly used adulticide, administered as an intramuscular injection (2.5 mg/kg) either in a two-dose (first intramuscular injection followed by the second 24 hr later) or three-dose (first injection followed by the two-dose protocol 4-6 weeks later) protocol. In order to minimize the likelihood of inducing potentially fatal pulmonary thromboembolism after the death of the heartworms, the dog must be placed on strict exercise restriction (i.e. cage rest) during and for 4-6 weeks after the treatment period. The three-dose protocol should be used in the treatment of dogs where there is significant risk of post-treatment pulmonary thromboembolism, however, current American Heartworm Society guidelines recommend the three-injection protocol be used in the treatment of all infected dogs [153]. Macrocyclic lactones also have shown some activity as adulticides, although the American Heartworm Society recommends that the *extra label *use of these compounds as a primary adulticide should be discouraged [153]. As an example, experimental trials have demonstrated that ivermectin has some efficacy as an adulticide if administered monthly at the preventive dosage of 6-12 mcg/kg for at least 16-30 months [154]. However, cardiopulmonary damage during this prolonged treatment period continues which may significantly affect the chances for a full clinical recovery [155,156]. In any case, in clinical cases which do not require an immediate treatment, the administration of a macrocyclic lactone for up to 6 months before administration of melarsomine can be beneficial. Such pre-treatment results in the reduction of adult worm mass and allows any immature worms present to fully mature at which time they are optimally susceptible to adulticide [92,157].

Use of the melarsomine adulticidal therapy in infected cats is considered an option only as a last resort due to the complexities of the infection in feline patients. Indeed, there is little data on the efficacy of melarsomine, which is not recommended in these animals due to a narrow therapeutic index. Studies have shown that melarsomine is toxic to cats at doses as low as 3.5 mg/kg [92,158,159]. Ivermectin has also shown some activity as an adulticide for use in cats. In particular, when administered *per os *at 24 mg/kg monthly for 24 months, the molecule may achieve a 65% reduction of worm burden [160].

A new strategy for the control of heartworms targets their *Wolbachia *bacterial endosymbionts. Treatment with tetracycline antibiotics has been reported to prevent larval development, inhibit embryogenesis in adult worms and eventually lead to the death of the adult stage of several filarial species [161,162]. Administration of both doxycycline and ivermectin for several months prior to or in the place of melarsomine treatment can eliminate adult worms with a reduced risk of thromboembolism. Thus, it is suggested that doxycycline has potential utility in the heartworm adulticide therapy for dogs and that a combination of doxycycline and ivermectin is adulticidal in dogs infected with *D. immitis *[92,163,164].

Given the severe pathogenic role of *D. immitis *heartworm, chemoprophylactic approaches are the best options for preventing the infection especially in endemic areas. The most widely used preventive drugs belong to the macrocyclic lactones class (avermectins and milbemycins), which are able to kill third and fourth larval stages of *D. immitis *[92]. Preventative protocols are based on the administration of anthelmintics once a month during the season of activity of vectors: oral ivermectin at 6 mcg/kg, milbemycin oxime at 500 mcg/kg and moxidectin at 3 mcg/kg, or topical selamectin at 6 mg/kg [92,160]. Regardless of the anthelmintic chosen, the administration should start within a month after the beginning of the mosquito season and finish within 1 month after the end of the season. Nevertheless, in endemic areas there is the recommendation to treat year-round with ivermectin, milbemycin oxime or selamectin, which provides protection when administered on a regular basis even if begun 3 months after infection [92,153,165]. In some countries there is also the option to use injectable moxidectin sustained-release formulation to protect dogs for the entire vector season [166,167].

The spot-on formulation containing imidacloprid 10%/moxidectin 1% may be successfully used for the prevention of *D. immitis *infection in cats [168].

## Conclusion remarks and future avenues

In conclusion, the purpose of this article was to provide an overview of current knowledge for the most important heartworm and lungworm infections of dogs and cats in Europe. In the past ten years the geographic range of these parasitic nematodes has expanded and new endemic foci in areas previously free or considered at minimal transmission risk has occurred. As a consequence, the number of reports of cardiopulmonary nematode infection in dogs and cats has greatly increased. There is a need to enhance the awareness of veterinary practitioners and diagnostic laboratories of the possibility of heartworm and lungworm infections, and to improve diagnostic means and control measures. Some copromicroscopic methods (e.g. Baermann technique) are not often applied in current practice, although cats and dogs may show clinical signs suggestive of parasitoses caused by cardiopulmonary worms. It is thus crucial that the Baermann method becomes a routine diagnostic test choice when dogs and cats present with signs suggestive of these infections [21,93]. Additionally, the Knott's method and antigen test kits are not used in those areas where *D. immitis *is erroneously thought to be absent. The key example of central-southern Italy in which the heartworm has recently been found should provide food for thought on these emerging issues [8,20,87]. In the coming years veterinarians will be faced more and more with problems and challenges related to the emergence of cardiopulmonary nematodes in companion animals. This is particularly true in those geographical regions which were considered free of infection and that, conversely, have become in the past few years infected with "new pathogens", e.g. *D. immitis *in the south and central regions of Italy and *A. vasorum *in Germany and The Netherlands. Additionally, several biological and epidemiological drivers, most of them yet to be elucidated, appear to be increasing the risk of infection in dogs and cats within the stable endemic foci of these parasites, as for instance *A. abstrusus *in Italy and the Iberian peninsula. Lack of awareness of these changing patterns have serious implications from a clinical standpoint, given that a delay in diagnosis and treatment can lead to severe lesions and even death in infected animals. The accurate diagnosis of these infections is necessary for the timely and effective treatment which can greatly impact the prognosis for dogs and cats affected by these parasites. Nonetheless, the lack of specificity of the clinical signs of infections caused by cardiopulmonary nematodes and the difficulties in the differential diagnosis, often lead to misdiagnosis, thus animals remain infected and untreated. Therefore, heartworm and lungworms should always be included in the differential diagnosis when pets are presented with cardiopulmonary disorders. Also, European veterinarians should be aware of diagnostic techniques available and of the current epidemiological situations, e.g. having always in mind that some nematodes (e.g. *A. vasorum*) are indeed emerging [7], some (e.g. *E. aerophilus*) are occasional but present and a potential danger for human health [20,36] and others (*Filaroides *and *Oslerus*) are almost absent and only sporadically reported in companion animals [169-171].

Given the impact heartworms and lungworms may have on animal health, the zoonotic potential of some of them and the trend in geographic spread, it is crucial that veterinary practitioners are aware of their importance and of appropriate diagnostic techniques and control plans.

## Competing interests

The authors declare that there are no competing interests and that the conceptual design, the content or any other scientific aspect have not been influenced.

## Authors' contributions

DT conceived the article and contributed to its drafting, preparation and intellectual content; ADC analysed the bibliographic data, processed the iconography and participated in drafting the article; GC contributed to the intellectual contents and inputs of the article and to its critical revision. All authors read and approved the final manuscript.
